# Supplementary figures and images for: Acute Influenza A virus outbreak in an enzootic infected sow herd: Impact on viral dynamics, genetic and antigenic variability and effect of maternally derived antibodies and vaccination
Source: PLoS One. 2019 Nov 14;14(11):e0224854. doi: 10.1371/journal.pone.0224854 (PMC6855628; doi:10.1371/journal.pone.0224854)

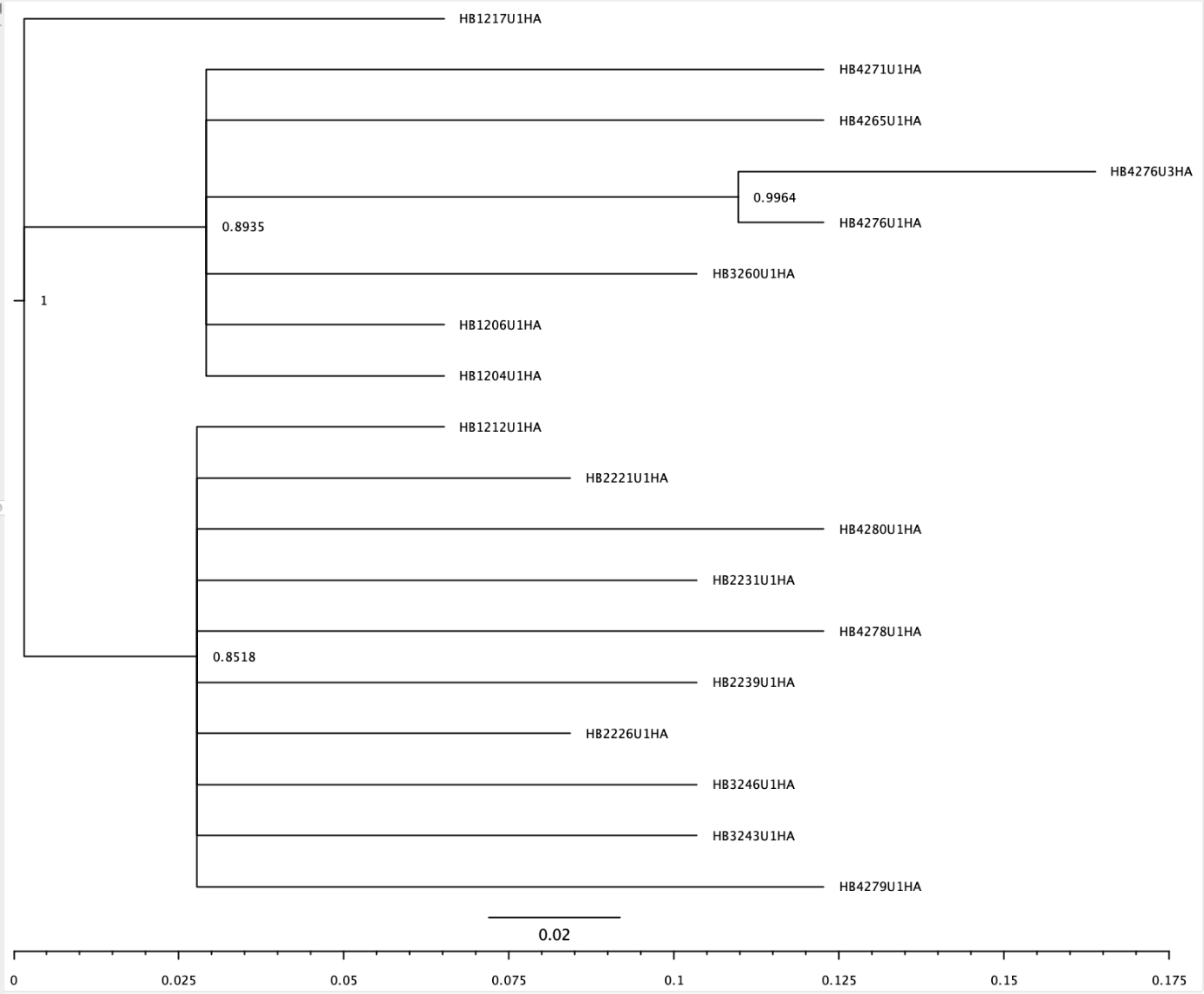

Supplement: S1 Fig — The x-axis represents time in years. Node labels represent posterior probabilities. The sequences are named as follows: HB indicates that the sequence was obtained in the first sampling round, and the following cipher gives the batch-number. The next three ciphers gives the ear tag number of the pig and “U1”, “U3”, “U5” and “U10” indicates the sampling time according to week 1, 3, 5 and 10–12. “HA” indicates that the sequences encode the hemagglutinin gene. The name of the sequences of the phylogenetic tree corresponds to the specific sequence ID “X” of the sequences uploaded in NCBI Genbank (A/sw/Denmark/X/2017(H1N2). (DOCX) [file pone.0224854.s001.docx]

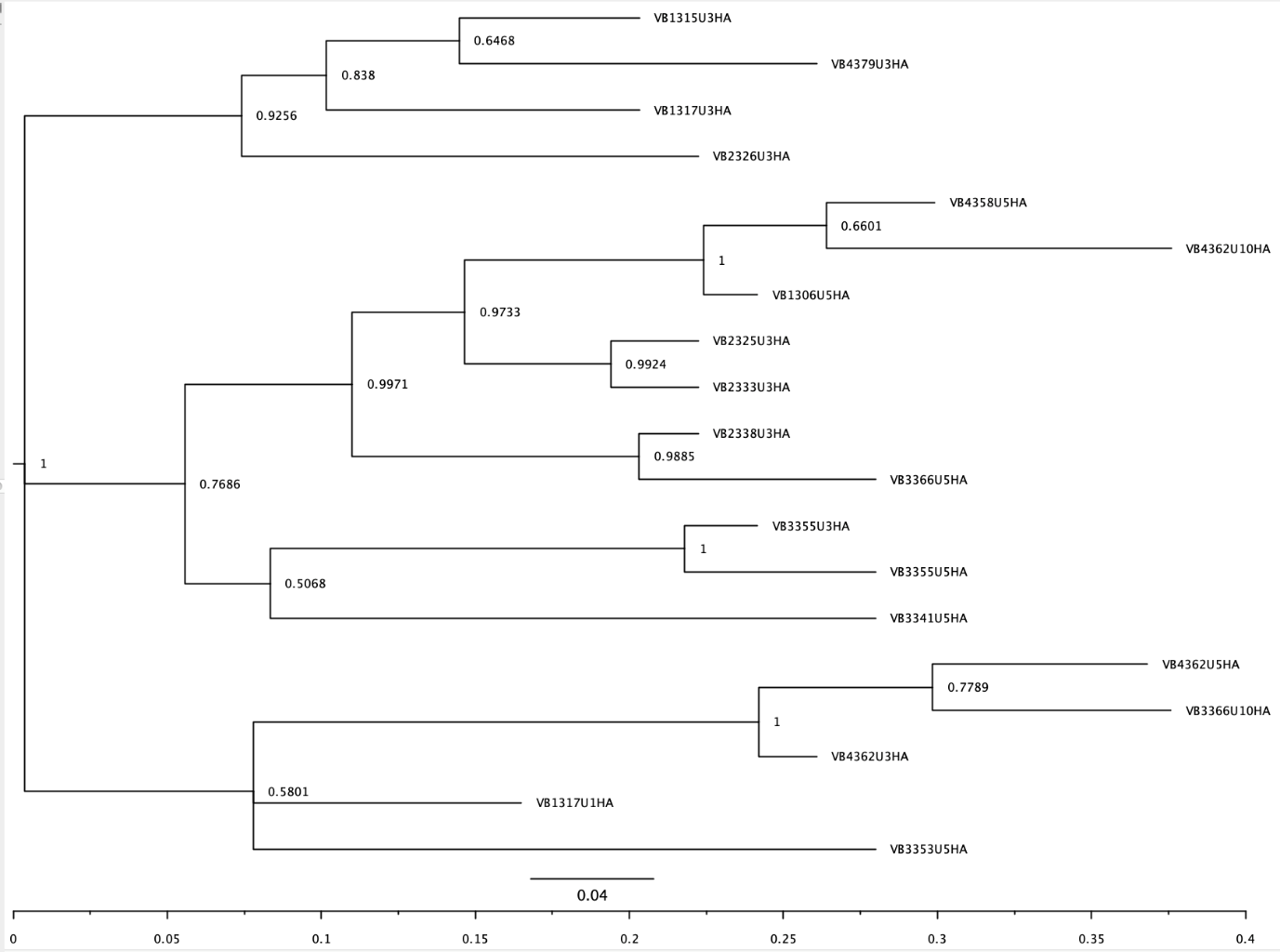

Supplement: S2 Fig — The x-axis represents time in years. Node labels represent posterior probabilities. The sequences are named as follows: VB indicates that the sequence was obtained in the second sampling round, and the following cipher gives the batch-number. The next three ciphers gives the ear tag number of the pig and “U1”, “U3”, “U5” and “U10” indicates the sampling time according to week 1, 3, 5 and 10–12. “HA” indicates that the sequences encode the hemagglutinin gene. The name of the sequences of the phylogenetic tree corresponds to the specific sequence ID “X” of the sequences uploaded in NCBI Genbank (A/sw/Denmark/X/2017(H1N2). (DOCX) [file pone.0224854.s002.docx]
