# Supplementary material for: Acute Influenza A virus outbreak in an enzootic infected sow herd: Impact on viral dynamics, genetic and antigenic variability and effect of maternally derived antibodies and vaccination
Source: PLoS One. 2019 Nov 14;14(11):e0224854. doi: 10.1371/journal.pone.0224854 (PMC6855628; doi:10.1371/journal.pone.0224854)
Supplement: S2 Table — The first columns describes the different genes. The second column describes the results of the pairwise comparison performed on the nucleotide consensus sequences. The third column describes the differences in amino acids according to the IUPAC codes. The forth column gives the position according to numbering from the first Methionine. The fifth column gives the number of sequences which had the given mutation compared to total number of sequences obtained from the samplings; 1st = 1st sampling and 2nd = 2nd sampling. (DOCX) [file pone.0224854.s004.docx]

| Gene: | Pairwise comparison (nt- difference): | Amino acid differences | Position | No. of seq: |
| --- | --- | --- | --- | --- |
| NA | 0-14 | I→T | 56 | 5/24 2nd |
|  |  | T→A | 71 | 1/24 2nd |
|  |  | K→R | 75 | 2/24 2nd |
|  |  | A→S | 88 | 1/24 2nd |
|  |  | K→E | 93 | 3/24 2nd |
|  |  | P→S | 340 | 1/24 2nd |
|  |  | G→C | 381 | 1/24 2nd |
|  |  | K→R | 403 | 2/24 2nd |
|  |  | G→V | 414 | 3/14 1st |
|  |  | G→S | 454 | 1/24 2nd |
| NS | 0-4 | A→S | 23 | 1/5 2nd |
|  |  | D→N | 24 | 1/5 1st |
|  |  | R→H | 59 | 1/5 1st |
| NP | 0-8 | S→G | 351 | 1/5 1st |
|  |  | M→L | 380 | 1/5 2nd |
| M | 0-4 | - | - | - |
| PB1 | 0-10 | I→V | 368 | 1/5 2nd |
|  |  | R→Q | 584 | 1/5 2nd |
|  |  | V→I | 724 | 5/5 2nd |
| PB2 | 0-7 | D→E | 60 | 1/5 2nd |
|  |  | R→S | 369 | 2/5 2nd |
| PA | 0-7 | M→I | 12 | 1/5 1st |
|  |  | I→V | 30 | 1/5 2nd |
|  |  | I→L | 118 | 1/5 1st and 5/5 2nd |
|  |  | I→V | 330 | 2/5 2nd |
|  |  | K→N | 360 | 1/5 2nd |
|  |  | V→I | 432 | 3/5 2nd |
|  |  | S→F | 709 | 1/5 1st |
