# Supplementary material for: Acute Influenza A virus outbreak in an enzootic infected sow herd: Impact on viral dynamics, genetic and antigenic variability and effect of maternally derived antibodies and vaccination
Source: PLoS One. 2019 Nov 14;14(11):e0224854. doi: 10.1371/journal.pone.0224854 (PMC6855628; doi:10.1371/journal.pone.0224854)
Supplement: Supplementary file 3 [file pone.0224854.s003.docx]

1st sampling round - before mass sow vaccination

	Batch 1				Batch 2				Batch 3				Batch 4			
	SOW 1	SOW 2	SOW 3	SOW 4	SOW 5	SOW 6	SOW 7	SOW 8	SOW 9	SOW 10	SOW 11	SOW 12	SOW 13	SOW 14	SOW 15	SOW 16
IAV-ELISA	POS	POS	POS	POS	POS	POS	POS	POS	POS	POS	POS	POS	POS	POS	POS	POS
HI-titer to P5-U4	320	320	40	80	1280	320	320	160	320	n/a	640	160	320	640	640	80
HI-titer to HB4	<20	<20	80	<20	<20	<20	<20	320	320	n/a	<20	640	<20	320	<20	160
HI-titer to VB4	<20	<20	<20	<20	<20	<20	<20	<20	320	n/a	<20	640	<20	640	<20	160
Week 1	201 + IAV	206 + IAV	211 + IAV	216 + IAV	221 + IAV	226 + IAV	231+ IAV	238	248	243 + IAV	250 + IAV	258	263 + IAV		273 + IAV	278 + IAV
	202 + IAV	207 + IAV	212 + IAV	217 + IAV		227 + IAV	232	239 + IAV	253		251 + IAV	259	264 + IAV	269 + IAV	274 + IAV	279 + IAV
	203 + IAV	208 + IAV	213 + IAV	218 + IAV	223 + IAV	228 + IAV	233 + IAV	240	254	245 + IAV	257 + IAV	260 + IAV	265 + IAV	270 + IAV	275 + IAV	280 + IAV
	204 + IAV	209	214 + IAV	219	224 + IAV	229	234	241	255	247 + IAV	246 + IAV	261 + IAV	266 + IAV	271 + IAV	276 + IAV	281 + IAV
	205	210	215 + IAV	220 + IAV	225 + IAV	230 + IAV	235 + IAV	242		249	256	262	267 + IAV	272 + IAV	277 + IAV	282 + IAV
Week 3	201	206	211	216	221	226	231	238	248	243	250	258	263		273	278
	202	207	212	217		227	232	239	253		251	259	264	269	274	279
		208	213	218	223	228	233	240	254	245	257	260	265	270	275	280
	204	209	214	219	224	229	234	241	255	247		261	266	271	276 + IAV	281
		210	215	220	225	230	235	242	252	249	256	262	267	272	277	282
Week 5	201	206	211		221	226	231	238	248	243	250	258	263		273	278
	202	207		217		227	232	239	253		251	259	264	269	274	279
	203	208	213	218	223	228	233	240	254	245	257	260	265	270	275	280
	204	209	214	219	224	229	234	241	255	247	246	261	266	271	276	281
	205	210	215	220	225	230	235	242	252	249	256	262	267	272	277	282
Week 10-12		206		216	221	226	231	238	248	243		258	263		273	
	202	207		217		227	232	239	253		251	259	264	269		279
	203	208	213	218		228	233	240	254	245		260	265	270	275	280
	204	209	214	219	224	229	234	241	255	247		261	266	271	276	281
	205	210	215	220	225	230	235	242	252			262	267	272	277	282

2nd sampling round - after mass sow vaccination

	Batch 1				Batch 2				Batch 3				Batch 4			
	SOW 1	SOW 2	SOW 3	SOW 4	SOW 5	SOW 6	SOW 7	SOW 8	SOW 9	SOW 10	SOW 11	SOW 12	SOW 13	SOW 14	SOW 15	SOW 16
IAV-ELISA	POS	POS	POS	POS	POS	POS	POS	POS	POS	POS	POS	POS	POS	POS	POS	POS
HI-titer to P5-U4	320	320	320	640	320	320	1280	80	640	1280	n/a	1280	1280	320	5120	1280
HI-titer to HB4	<20	320	320	320	320	<20	320	<20	320	160	n/a	640	640	<20	640	320
HI-titer to VB4	<20	320	160	<20	160	<20	1280	<20	160	160	n/a	<20	320	<20	640	160
**Week 1**	301	306	311	316	326	321	331	336	**341 + IAV**	346	351	366		361	371	376
	302	307	312	**317 + IAV**	327	322	332	337	342	347	352		357	362	372	377
	**303 + IAV**	308	313	**318 + IAV**	328	323	333	338	343	348	353	368	358		373	378
	**304 + IAV**	309	314	319	329	324	334	339	**344 + IAV**	349	354	369	359	364	374	379
	305	310	315		330	325	335	340	**345 + IAV**	350	355	370		365	375	380
**Week 3**	301	306	311	316	**326 + IAV**	**321 + IAV**	**331 + IAV**	**336 + IAV**	341	346	**351 + IAV**			**361 + IAV**	371	376
	302	307	**312 + IAV**	**317 + IAV**	**327 + IAV**	322	**332 + IAV**	**337 + IAV**	342	347	**352 + IAV**		**357 + IAV**	**362 + IAV**	372	377
	303	308	**313 + IAV**	318	328	**323 + IAV**	**333 + IAV**	**338 + IAV**	343	348	**353 + IAV**	**368 + IAV**	**358 + IAV**		373	**378 + IAV**
	304		314	319	329	324	**334 + IAV**	**339 + IAV**	344	349	**354 + IAV**	369	**359 + IAV**		374	**379 + IAV**
	305	310	**315 + IAV**		330	**325 + IAV**	**335 + IAV**	**340 + IAV**	345	350	**355 + IAV**	370			375	380
**Week 5**	**301 + IAV**	**306 + IAV**	311	316	**326 + IAV**	321	331	336	**341 + IAV**	**346 + IAV**	**351 + IAV**	**366 + IAV**		**361 + IAV**	**371 + IAV**	**376 + IAV**
	**302 + IAV**	**307 + IAV**	312	317	327	322	332	337	**342 + IAV**	347	**352 + IAV**		357	**362 + IAV**	372	**377 + IAV**
	**303 + IAV**	**308 + IAV**	313	318	328	**323 + IAV**	333	338	**343 + IAV**	348	**353 + IAV**	**368 + IAV**	**358 + IAV**		**373 + IAV**	**378 + IAV**
	**304 + IAV**		314	319	**329 + IAV**	**324 + IAV**	334	339	**344 + IAV**	**349 + IAV**	354	369	**359 + IAV**		**374 + IAV**	**379 + IAV**
	305	**310 + IAV**	315		330	325	335	340	**345 + IAV**	**350 + IAV**	**355 + IAV**	370	360	**365 + IAV**	**375 + IAV**	**380 + IAV**
**Week 10-12**	301	306	311	316	**326 + IAV**	**321 + IAV**	331	336	341	346	351	**366 + IAV**		**361 + IAV**	371	
	302	307	312	317	327	**322 + IAV**	332	337	342	347	352		357	**362 + IAV**	372	377
	[illegible]	[illegible]	[illegible]	[illegible]	[illegible]	[illegible]	[illegible]	[illegible]	[illegible]	[illegible]	[illegible]	[illegible]	[illegible]		[illegible]	[illegible]
